# Influence of Oxygen Content and Microstructure on the Mechanical Properties and Biocompatibility of Ti–15 wt%Mo Alloy Used for Biomedical Applications

**DOI:** 10.3390/ma7010232

**Published:** 2014-01-06

**Authors:** José R. S. Martins, Raul O. Araújo, Tatiani A. G. Donato, Victor E. Arana-Chavez, Marília A. R. Buzalaf, Carlos R. Grandini

**Affiliations:** 1 UNESP—Univ. Estadual Paulista, Laboratório de Anelasticidade e Biomateriais, 17.033-360, Bauru, SP, Brazil; E-Mails: jrsmjr@fc.unesp.br (J.R.S.M.J.); raularaujo@fc.unesp.br (R.O.A.); 2 USP—Universidade de São Paulo, Faculdade de Odontologia, Departamento de Biomateriais e Biologia Oral, 05.508-900, São Paulo, SP, Brazil; E-Mails: tatianidonato@gmail.com (T.A.G.D.); vearana@usp.br (V.E.A.-C.); 3 USP—Universidade de São Paulo, Faculdade de Odontologia de Bauru, Departamento de Ciências Biológicas, 17.012-901, Bauru, SP, Brazil; E-Mail: mbuzalaf@fob.usp.br

**Keywords:** Ti alloys, microstructure, Rietveld’s method, modulus of elasticity, biocompatibility

## Abstract

The Ti–15Mo alloy has its mechanical properties strongly altered by heat treatments and by addition of interstitial elements, such as, oxygen, for example. In this sense, the objective of this paper is to analyze the effect of the introduction of oxygen in selected mechanical properties and the biocompatibility of Ti–15Mo alloy. The samples used in this study were prepared by arc-melting and characterized by density measurements, X-ray diffraction, scanning electron microscopy, microhardness, modulus of elasticity, and biocompatibility tests. Hardness measurements were shown to be sensitive to concentration of oxygen. The modulus results showed interstitial influence in value; this was verified under several conditions to which the samples were exposed. Cytotoxicity tests conducted *in vitro* showed that the various processing conditions did not alter the biocompatibility of the material.

## 1. Introduction

The area of biomaterials has always taken into consideration the appropriate combination between physical properties adjacent to the tissue replaced and minimizing toxic response to the foreign body. One set of materials that presents this favorable combination is titanium and its alloys, which have been widely used in the manufacture of prostheses and special devices in the medical and dental areas since 1970, due to such properties as low values of Young’s modulus, corrosion resistance, and biocompatibility characteristics [1].

Currently, Ti–6Al–4V alloy is widely used as a structural biomaterial for dental and orthopedic implants, as it has excellent mechanical strength and corrosion resistance. However, studies show that release of aluminum and vanadium ions from the alloy can cause health problems in the long term. It is well known that aluminum ions cause neurological disorders [2] and vanadium ions are associated with enzymatic problems, among others [3].

Due to this, there is a need to obtain alloys with mechanical and electrochemical properties better than Ti–6Al–4V alloys and free of aluminum and vanadium. Titanium-based alloys that are most promising are those that present niobium, zirconium, tantalum, and molybdenum as alloying elements [4].

Research on new titanium-based alloys focuses on β type alloys (with bcc crystalline structure), as the processing variables can be controlled to produce the required results. The best properties, such as lower modulus of elasticity, increased corrosion resistance, and better response of bone tissue, are possible when compared with alloy types α and α + β [4,5].

Ho *et al.* [6] studied a series of Ti–Mo binary alloys, using techniques, such as X-ray diffraction, optical microscopy, microhardness, flexural strength, and modulus of elasticity, showing that the microstructure is dependent on molybdenum concentration. From 3 wt% to 5 wt% of molybdenum shows a hexagonal compact crystalline structure (α′ phase); with 6 wt% shows, simultaneously, hexagonal compact and orthorhombic crystalline structure (α′′ phase); with 7.5 wt%, only orthorhombic crystalline structure is shown; with 9 wt%, orthorhombic and body-centered cubic (β phase) crystalline structures coexist and, over 10 wt%, only body-centered cubic crystalline structure is shown. The other analyzed properties are dependent on the microstructure; for example, the modulus of elasticity is smaller in the β phase.

Sukedai *et al.* [7] studied in Ti–20Mo the role of vacancies, the formation of clusters, and the relationship of these defects with the β phase transformation for ω, a metastable phase with crystalline structure that may be trigonal or hexagonal. To do so, these authors used transmission electron microscopy techniques, similar to that carried out by Guo and Enomoto [8], concluding that phase ω fragilizes the material.

Chen *et al.* [9] studied the microstructure and mechanical properties of Ti–Mo system alloys containing 5 wt%, 10 wt%, 15 wt%, and 20 wt% of molybdenum by means of X-ray diffraction measurements and also showed that the crystalline structure and microstructure are dependent on molybdenum content. For mechanical properties, such as microhardness, elastic modulus, and compression resistance, it is observed by the authors that these properties decrease with molybdenum content, except for microhardness, where the reverse occurs.

Oliveira *et al.* [10–12] studied a set of binary alloys of Ti–Mo, using microstructural analysis and electrochemistry characterization. Microstructural analysis shows similar results to Ho *et al.* [6] and electrochemical characterization results show good corrosion resistance in all studied alloys; molybdenum has a fundamental role in improving the protection characteristics of its spontaneous oxides.

Among the Ti–Mo system alloys, Ti–15Mo is highlighted for its excellent corrosion resistance and good combination of mechanical properties, such as fatigue, hardness, wear resistance, *etc.* This alloy, with good properties for biomedical applications, is already included by the American Society for Testing and Materials [13].

Niinomi [5] studies modulus of elasticity, fatigue, and mechanical strength for various titanium alloys, among them Ti–15Mo. This author also analyzes the simulated environment of the human body on mechanical properties, where the alloys were implanted in rabbits for 11 months, showing that the long-term usage of biomaterials, such factors as Young’s modulus, functionalities, such as superelasticity and shape memory characteristics, and mechanical properties, including fatigue characteristics, fretting fatigue characteristics, fracture toughness, and wear characteristics, are considered critical.

Martins Jr. *et al.* [14] prepared Ti–15Mo alloy using arc-melting and measured modulus of elasticity, microhardness, and biocompatibility, observing that this alloy has smaller modulus and greater microhardness than cp–Ti. In addition, these authors performed indirect cytotoxicity assays and cell viability, showing satisfactory response from the alloy.

An analysis of the relationship between the phases and the microstructure of Ti–15Mo on six processing conditions was made by Martins Jr. and Grandini [15], using X-ray diffraction measurements and analysis of the diffractograms using the Rietveld method. There were variations in the intensity of the peaks, which were attributed to increased concentration of α′ phase. Variations were also observed in crystallite size and crystalline lattice microstrains, leading to the conclusion that homogenization heat treatment decreased the microstrains and increased crystallite size.

In another study, Martins Jr. *et al.* [16] evaluated diffusion of oxygen and nitrogen in Ti–15Mo alloys by mechanical spectroscopy measurements, obtaining the diffusion coefficient and activation energy for these elements under various processing conditions.

This paper aims to evaluate the effect of heat treatments and the introduction of oxygen on structure, microstructure, selected mechanical properties, and biocompatibility of Ti–15Mo alloy.

## 2. Experimental Procedure

The samples used in this study are titanium alloys containing 15% by weight of molybdenum, produced by arc-melting, in the Laboratório de Anelasticidade e Biomateriais, UNESP/Bauru, Brazil [14]. Characterization of the samples was carried out by means of quantitative chemical analysis, density, X-ray diffraction, scanning electron microscopy, microhardness, and modulus of elasticity measurements. The samples were analyzed after melting and after homogenization heat treatment. More details on chemical, structural, and microstructural characterization are shown in Martins Jr. *et al.* [14–16].

After homogenization heat treatment, the samples were subjected to a gas doping process, conducted in a quartz tube under a vacuum of 10^−7^ mbar, heating rate of 10 K/min and plateau of 973 K by 2.0 h, and quick cooling with water to room temperature. The partial pressures of oxygen introduced into the quartz tube were 5 × 10^−1^ Torr (sample Ti–15Mo#3), 1.6 × 10^−2^ Torr (sample Ti–15Mo#4), and 1.6 Torr (sample Ti–15Mo#5). To measure the amount of oxygen in the material, gas analysis was performed by thermoconductivity difference using a LECO model TC-400 equipment. It is very important to analyze the influence of interstitials elements, such as carbon, hydrogen, and nitrogen, but oxygen is of particular interest here, as it can be present in condensed phases or interstitially dissolved in solid solution within the metallic matrix [17].

Density measurements were obtained via an Explorer model Ohaus precision balance and density determination kit, which is based on Archimedes’ principle. Each density value represents the average of five measurements.

Microhardness measurements were performed via a Shimadzu model HMV-2, using a load of 200 gf (1.96 N) by 60 s. For each sample, five measurements were made in different positions. The values of microhardness tests match the average of 10 measurements.

Dynamic modulus of elasticity was obtained by mechanical spectroscopy measurement using the torsion pendulum technique operating in the frequency range 4–30 Hz, in which it is possible to measure damping of the free vibrations of the system, which are related to elastic energy loss (internal friction) and the oscillation frequency, *f*, associated with the elasticity modulus, *E* [18,19].

To verify the biocompatibility of the prepared samples, *in vitro* cytotoxicity tests—MTT modified and analysis via scanning electron microscope (SEM)—were conducted. For these tests, osteogenic cells of MC3T3-E1 lineage (pre-osteoblasts from thickened calvarium mouse newborns-ATCC) were utilized; they were cultivated on the titanium alloy for 72 h (MTT test) and 24 h (SEM analysis). Eight samples of Ti–15Mo were used after each processing condition, based on ISO 10993-5 standard [20], with a diameter of 4 mm and a thickness of 1 mm; they were weighed so that the amounts were calculated from the culture medium MEM-α, required for the direct cytotoxicity test.

The modified MTT15 assesses the reduction ability of tetrazolium salt (3-[4,5-Dimethylthiazol-2-yl]-2,5-Diphenyltetrazolium Bromide) (MTT, 5 mg·mL^−1^ Phosphate buffered saline (PBS)—Sigma-Aldrich, St. Louis, MO, USA) by the mitochondrial enzyme *Succinato desidrogenase* to fromazan crystals at the end of the reaction. As the MTT is a colorimetric test, the yellow MTT solution changes to purple when it reacts with the mitochondrial enzyme. The intensity of this reaction can be quantified by a spectrophotometer reading that measures absorbance at 562 nm using a microplate reader. For testing direct cytotoxicity, the cells were plated under the surface of the material. A polystyrene (coverslip) was used as the negative control, and a solution of α-MEM, 10% of FBS, and 1% of phenol was used as the positive control.

MTT analysis was carried out in triplicate with *n* = 5. The results were analyzed statistically by ANOVA test complemented with Tukey’s test. The level of significance was 5% (*p* ≤ 0.05). To corroborate the results of cell viability, the osteoblasts were cultivated on the alloy for 24 h. After 24 h, the cells were fixed in 4% paraformaldehyde +0.1% glutaraldehyde buffered with sodium cacodylate, pH 7.2, for 1 h, at room temperature and routinely processed for scanning electron microscopy. Samples were examined post-fixed with 1% osmium tetroxide, dehydrated in crescent concentrations of ethanol, and desiccated after treatment with HMDS.

Specimens were mounted onto aluminum stubs, sputtered with gold, and examined in a JEOL 6100 scanning electron microscope. Cells grown on a glass coverslip were used as negative control [21].

## 3. Results and Discussion

Chemical analysis of as-cast Ti–15Mo alloys shows that the molybdenum concentration is 14.47 wt%, which is in accordance with ASTM F 2066-0816, which establishes a tolerance of 14 wt% to 16 wt% of molybdenum [14]. Energy dispersive spectroscopy (EDS) mapping shows homogenous distribution of alloying elements rather than formation of segregated regions, *i.e.*, regions with the largest concentrations of a given element [15]. The samples were doped with oxygen from 0.13 wt% to 0.25 wt% [15]. Maximum nitrogen content was 0.02 wt%, in accordance with the ASTM F 2066-0816, which establishes (0.05 ± 0.02) wt% as the maximum level tolerated for nitrogen.

Structural characterization of the samples made by X-ray diffraction shows peaks characteristic of a body-centered cubic structure, which is typical of these β phase alloys and α′ phase that has hexagonal compact crystalline structure; however, this last phase is a martensitic type phase, *i.e.*, it is a metastable phase formed by rapid cooling, leaving no time to accommodate a more stable structure [15]. The diffractograms were analyzed by the Rietveld method and it was possible to quantify the volumetric fraction of crystalline phases present. After analysis, it was found that the variation of the intensities of the peaks, associated with the increase in concentration of α′ phase, increased with the oxygen content. Microstructural analysis of the samples after oxygen doping showed a successive increase in the amount of the α′ phase, which can be explained by the fact that the sample was heated to 700 °C and underwent rapid cooling, thus, retaining the α′ phase [15].

The values obtained for alloy density in the various conditions are shown in Table 1.

The values obtained for the density of Ti–15Mo#0 coincide with the theoretical value of Ti–15Mo [22], which indicates that the stoichiometry was respected and reinforces the fact that the produced samples are of good quality. After swaging (Ti–15Mo#1), there was an increase in density, which may be explained by microstructural change with increasing α′ phase, since this phase has a higher density in relation to β phase, due to higher packaging factor. In samples Ti–15Mo#2, Ti–15Mo#3, and Ti–15Mo#5, the densities are equal and are higher than Ti–15Mo#0, which is also connected with microstructural change of these samples caused by α′ phase. In sample Ti–15Mo#4, there was a further increase in density, which can be explained by microstructural change given that the amount of phase α′ is near the Ti–15Mo#1 sample, as shown in Table 1. Although the samples have oxygen and nitrogen, these gases do not influence the value of the density, which can be explained by the fact that the densities of oxygen and nitrogen, 1.42 × 10^−3^ g/cm^3^ and 1.25 × 10^−3^ g/cm^3^, respectively, are much smaller when compared with the density alloy elements, molybdenum, and titanium, with 10.22 g/cm^3^ and 4.54 g/cm^3^, respectively. Thus, the interstitial elements do not influence significantly the density of the alloys.

**Figure 1.**
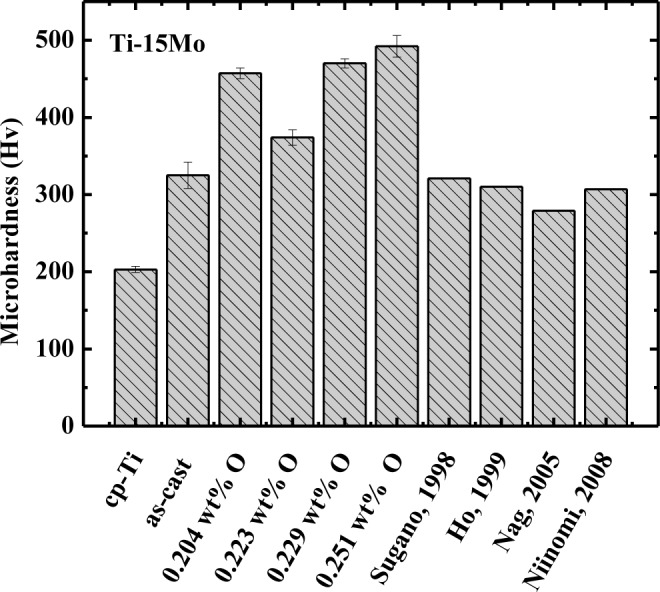
Vickers microhardness values for Ti–15Mo samples, compared with literature results.

The values of the Vickers microhardness testing for Ti–15Mo samples containing oxygen are shown in Figure 1, which also shows several previously published results from the literature. From the obtained values, it is possible to observe that the addition of molybdenum increases the microhardness value in relation to cp–Ti, which is expected, as molybdenum has greater hardness than titanium. It can be verified that the hardness value of as-cast Ti–15Mo alloy is in accordance with values published in the literature [5,6,23,24], and, with oxygen doping, there is a significant increase in hardness values.

Following heat treatment (sample Ti–15Mo# 2), there was a decrease in α′ phase, and it was expected that the hardness had lessened, but there was no change, which is possibly due to the increase in oxygen concentration. As oxygen is very reactive, it may have formed a solid solution and the solid solution hardened, which compensates for the decrease in α′ phase. In this way, all the oxygen atoms in the sample are not interstitial; a part may have formed oxide and caused alloy hardening. With the first doping on oxygen (sample Ti–15Mo#3), there was a significant increase in hardness, which can be explained by the fact of α′ phase having been increased and the oxygen concentration being the highest of all conditions; solid solution hardening also may have occurred. This assertion is corroborated by mechanical spectroscopy results [16], once for the Ti–15Mo#3; although it has the highest oxygen content, the decomposition of relaxation peak shows that the Ti–O and Mo–O processes have little intensity, *i.e.*, few oxygen atom occupy the interstitial sites, to cause stress-induced ordering of oxygen atoms around titanium and molybdenum.

In metals with body centered cubic crystalline structure, point defects as heavy interstitial solute atoms are preferably positioned in octahedral sites, causing local deformation of the crystalline lattice with tetragonal symmetry. In the absence of an external mechanical stress, interstitial atoms are randomly distributed in the tetragonal sites. With the application of an external mechanical stress occurs the reorientation of interstitial atoms to equivalent sites. This reorientation will depend on the time, *i.e.*, these processes cause a change with the time of the balance of the defects configuration for a new and unique equilibrium state, when the mechanical external stress is applied. When this stress is removed the change is rolled back, and, over time, the original equilibrium state is restored. The dissipation of energy generated during the reorientation of the interstitial solutes, called internal friction, reaches a maximum value in a characteristic temperature (depending on frequency), known as Snoek’s peak [17,25].

After the second and third oxygen doping, Ti–15Mo#4 and Ti–15Mo#5 samples, respectively, there was a decrease in hardness compared to Ti–15Mo#3. Although the concentration of α′ phase increased for Ti–15Mo#4 and Ti–15Mo#5 samples, it would be expected to increase hardness, but a decrease occurred. A possible explanation for this observation is that the oxygen has the role of reducing hardness when it is interstitial, which is corroborated by mechanical spectroscopy measures, as Ti–O and Mo–O processes are quite intense [16]. Thus, it can be observed that the behavior of microhardness on the basis of the quantity of oxygen and α′ phase analyzed independently differs, which means it must be analyzed simultaneously, as each stage of processing varies the microstructure and the oxygen concentration.

**Figure 2.**
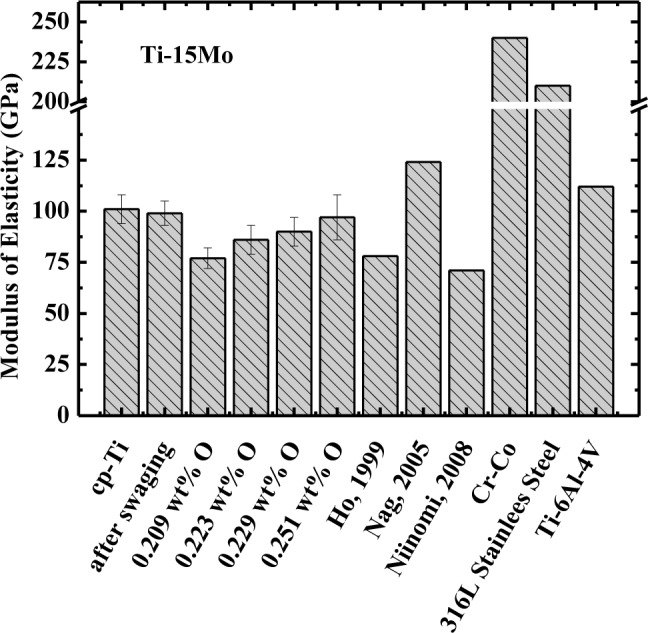
Modulus of elasticity values for Ti–15Mo samples used in this study, compared with literature results.

The modulus of elasticity (obtained at 37 °C, body temperature) of commercially pure titanium alloy, Ti–15Mo after swaging (Ti–15Mo#1 sample), after heat treatment (Ti–15Mo#2 sample), and after oxygen doping (Ti–15Mo#3, Ti–15Mo#4, and Ti–15Mo#5 sample), is presented in Figure 2. It is evident that the value of modulus of Ti–15Mo samples used in this study is in accordance with the previous results obtained in the literature [5,6,24]. The values of the modulus for Ti–15Mo#1, Ti–15Mo#3, and Ti–15Mo#4 samples showed the same values of cp–Ti, within the uncertainty of measurements. However, for Ti–15Mo#2 and Ti–15Mo#5 samples, the values were about 10% smaller than the cp–Ti, even taking into account measurement uncertainty. In any case, the modulus values are below other materials, such as Ti–6Al–4V alloy, which is one of the most commonly used in the orthopedic area, as well as the values for 316L stainless steel and Co–Cr.

It is well known from the literature that the modulus of elasticity for phase α′ shows a value higher than the value for the β phase. The modulus of elasticity is related to the attraction and repulsion forces between atoms, and as the equilibrium distance between atoms increases, the value of the modulus tends to decrease. Comparing the crystalline structure of phases α′ and β, it is evident that the distance between the atoms is higher in body-centered cubic structure (β phase). With the atoms closer together, the attraction force is higher and thus an increase in the value of the modulus of elasticity occurs [26].

It can be shown that addition of molybdenum decreases the value of the modulus of elasticity in relation to cp–Ti, which is consistent with the literature [5,6,24]. Analyzing the various processing conditions of Ti–15Mo samples, it is possible to observe that the Ti–15Mo#1 sample has the highest modulus of elasticity (99 GPa) and this can be explained by the large amount of phase α′ present in the microstructure, and internal stresses caused by swaging. In the sample following homogenization heat treatment (Ti–15Mo#2 sample), there was a significant decrease in the value of the modulus of elasticity (around 11%). This is associated with decreased amount of α′ phase and with relief of internal stresses arising from the swaging. The α′ phase possesses hexagonal compact crystalline structure, given that this type of structure has high packaging factor (0.74) (*i.e.*, atoms are closer and the strength of the chemical bond between them is more intense). The modulus of elasticity is closely connected with the nature of the chemical bonds, thus increases in the intensity of the chemical bonding module will be higher. After the first doping on oxygen (Ti–15Mo#3 sample), there is an increase in modulus, which can be explained by the fact that the α′ phase increased, as well as the amount of oxygen. Oxygen undergoes rearrangement of the position of its atoms in solid solution in phase β, leading to an increase in modulus of elasticity. Thus, as occurred in the hardness analysis, the relationship between the microstructure and the oxygen content should be considered simultaneously, showing that changing the value of the modulus is interrelated with these two parameters.

The results show that this material has great potential to be used as a biomaterial, mainly in orthopedic applications. The values of the modulus of elasticity are much smaller when compared to cp-Ti and titanium alloys of type α and α + β. However, these values are far superior to that of bone, generally between 17 and 28 GPa [4].

**Figure 3.**
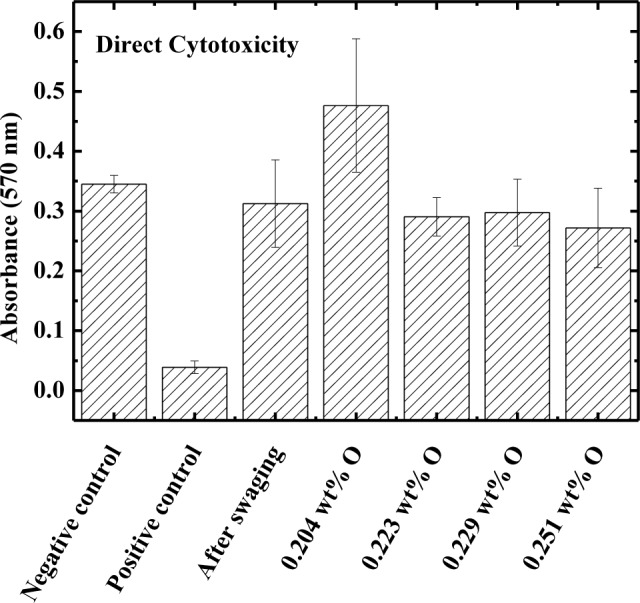
Direct cytotoxicity for Ti–15Mo samples in all conditions.

The direct cytotoxicity test results are shown in Figure 3 for the Ti–15Mo samples after swaging, after heat treatment, and after oxygen doping. Phenol was used as positive control and the culture dish was used as negative control. The results show that the absorbance values for the samples of Ti–15Mo under all conditions studied are closer to the negative control, *i.e.*, negative for cytotoxicity. In this way, the various processing conditions do not affect biocompatibility [27].

**Figure 4.**
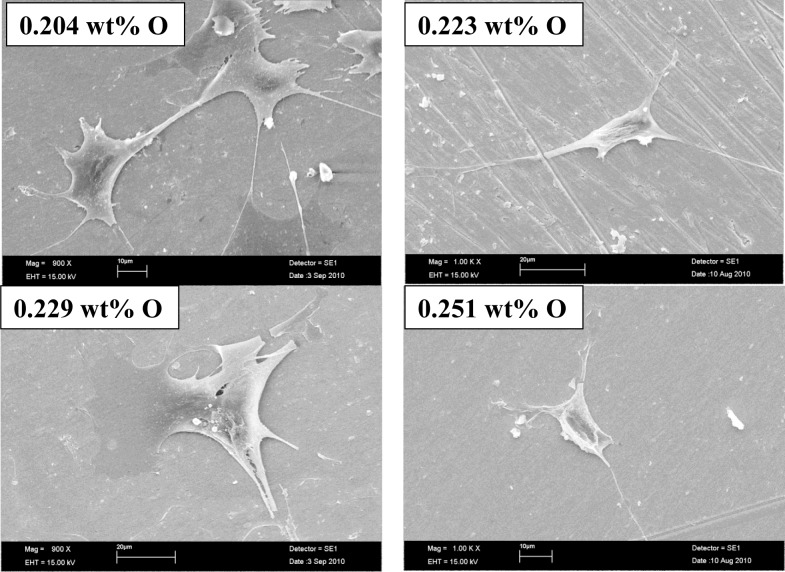
Scanning electron micrographs of cells grown on Ti–15Mo alloy samples doped with four quantities of oxygen.

Figure 4 shows images of scanning electron microscopy of osteoblastic cells on Ti–15Mo samples in all processing conditions used. It is evident that osteoblastic cells possess a prolonged morphology and the release of the cell cytoplasm, suggesting good fixation on the surface of the samples, which shows good integration with the study material, revealing that the material does not cause cytotoxic effects, which is very desirable in materials to be used as biomaterials [27–29].

## 4. Conclusions

Hardness measurements show that the alloy has higher hardness compared to cp–Ti. The results are sensitive to the concentration of oxygen and the alloy microstructure, thus, they are interdependent.

The modulus results show that the microstructure and the concentration of interstitial influence its value; it is possible to verify this along the several conditions to which the samples were subjected. The modulus of elasticity found for this alloy varies from about 86 to 100 GPa. These values are close to the values found in the literature for β type titanium alloys.

Biocompatibility tests conducted *in vitro* show results closer to the negative control, showing that the material is not cytotoxic. With scanning electron microscopy images, it was possible to show the cell morphology, observe cellular fixation and development; the various processing conditions do not alter the biocompatibility of the material.

## Figures and Tables

**Table 1.** Density and fraction of α′ phase for Ti–15Mo samples.

Sample	Oxygen (wt%)	Nitrogen (wt%)	ρ (g/cm^3^)	α′ phase (%)
Ti–15Mo#0	0.137 ± 0.005	0.006 ± 0.001	4.96 ± 0.01	2.3
Ti–15Mo#1	0.177 ± 0.004	0.007 ± 0.002	5.02 ± 0.01	24.5
Ti–15Mo#2	0.223 ± 0.002	0.001 ± 0.002	4.99 ± 0.01	2.1
Ti–15Mo#3	0.251 ± 0.008	0.020 ± 0.002	4.98 ± 0.01	4.3
Ti–15Mo#4	0.229 ± 0.005	0.018 ± 0.003	5.03 ± 0.02	18.2
Ti–15Mo#5	0.204 ± 0.008	0.015 ± 0.003	5.00 ± 0.01	27.1
Ti–15Mo [22]	–	–	4.97	–
cp–Ti	–	–	4.50 ± 0.01	–
